# A pilot study: lenticule quality of hyperopic small incision lenticule extraction (SMILE) in rabbits

**DOI:** 10.1186/s12886-020-01432-x

**Published:** 2020-04-19

**Authors:** Yu Zhao, Feng Zhao, Tian Han, Jing Zhao, Xingtao Zhou

**Affiliations:** 1 Department of Ophthalmology, Eye and ENT Hospital of Fudan University, 83 Fenyang Road, Shanghai, 200031 PR China; 2 NHC Key Laboratory of Myopia (Fudan University); Laboratory of Myopia, Chinese Academy of Medical Sciences, Shanghai, China; 3 Shanghai Research Center of Ophthalmology and Optometry, Shanghai, China; 4 Department of Ophthalmology, Shuguang Hospital Affiliated to Shanghai University of Traditional Chinese Medicine, Shanghai, China

**Keywords:** Hyperopia correction, Lenticule quality, Small incicion lenticule extraction, Refractive surgery

## Abstract

**Background:**

To evaluate lenticule surface characteristics of small incision lenticule extraction (SMILE) for hyperopia correction in rabbits.

**Methods:**

The left and right eyes of 8 rabbits were divided into two groups. The right eyes were assigned to a myopia group, and the left eyes to a hyperopia group. The rabbits received SMILE procedures with + 3.00 D and − 3.00 D correction for the hyperopia and myopia groups, respectively. Extracted lenticules were examined via scanning electron microscopy. Lenticules from odd-numbered rabbits were accessed with the anterior surface, and lenticules from even-numbered rabbits were observed with the posterior surface. A previously established scoring system was used to evaluate lenticule surface characteristics. Statistical analysis was conducted to compare the scores between the two groups.

**Results:**

All procedures were performed successfully, and the lenticules were extracted smoothly. One myopia lenticule that was facing downward was handled failed in preparation for imaging, thus 15 lenticules were ultimately graded. Twelve lenticules exhibited smooth surfaces, and regularly arranged tissue bridges were observed in almost all regions. Three lenticules exhibited a partially rough surface and irregularities affecting more than 10% of the lenticules (2 in the hyperopia group and 1 in the myopia group). Rough lenticules occurred in twice as many lenticules in the hyperopia group compared to the myopia group.

**Conclusions:**

Scan quality of lenticules after SMILE for hyperopia correction is comparable to that of myopia lenticules. The shape of hyperopic lenticule may increase the difficulty of surgical manipulation and result in surface roughness.

## Synopsis

The shape of hyperopic lenticule may increase the difficulty of surgical manipulation and result in surface roughness.

## Background

The surgical correction of hyperopia presents a greater challenge than that of myopia for multiple reasons [1, 2]. Researchers have studied different refractive techniques for correcting hyperopia, including laser epithelial keratomileusis (LASEK), laser in situ keratomileusis (LASIK), and femtosecond LASIK. These laser platforms have already demonstrated satisfactory predictability and stability of hyperopia correction [1, 3].

Small incision lenticule extraction (SMILE) using a femtosecond laser is a well-established surgical procedure for correcting myopia [4]. Thus, the feasibility of using SMILE for hyperopia correction has attracted increased research attention in the field of refraction surgery [5, 6]. Considering that lenticule surface quality is vital for postoperative optical quality, the purpose of the current study was to evaluate the surface quality of rabbit corneal lenticules extracted during SMILE for hyperopia correction [7].

## Methods

### Animals

Eight healthy adult male New Zealand rabbits (provided by the Department of Animal affiliated with Shanghai Medical College of Fudan University) weighing between 2.5 and 3.0 kg and free of anterior segmental diseases were used in the study. The left eyes of all rabbits were assigned to a hyperopia group, and the right eyes to a myopia group. All the experimental and animal handling procedures met the tenets of the Association for Research in Vision and Ophthalmology Statement for the Use of Animals in Ophthalmic and Vision Research, and adhered to the requirements of the Animal Research and Ethics Committee of the Eye and ENT Hospital, Fudan University, Shanghai, China who also approved this study.

### Surgical technique

All surgical procedures were performed by the same surgeon under general anesthesia with an intramuscular injection of ketamine hydrochloride (50 mg/kg) and xylazil (5 mg/kg). The VisuMax femtosecond laser system (Carl Zeiss Meditec AG, Berlin, Germany) was used to perform SMILE and the same surgeon (XZ) performed all procedures. Rabbits were anesthetized via the intramuscular injection of ketamine hydrochloride (125 mg/kg) and topical anesthesia with 0.4% oxybuprocaine hydrochloride eye drops. The femtosecond laser was set at a repetition rate of 500 kHz and a pulse energy of 130 nJ. A small (S) size contact glass was used in all myopia cases and a medium (M) size was applied for hyperopia correction. The suction ring was activated to fix the eyeball prior to laser scanning. A corneal cap of 7.5 mm with a thickness of 110 μm was applied. Lenticule diameter was set as 5.5 mm. The length of the side cut was set to a 2-mm angle at the superior 12-o’clock position. The intended hyperopia and myopia correction was + 3.00 and − 3.00 diopters spheres, respectively. The central minimum lenticule thickness of hyperopia lenticule was 25 μm, and a transition zone was 0.2 mm. After scanning was completed, the refractive lenticule was separated and removed through the side cut incision smoothly. The rabbits were kept for further studies (wound healing response).

### Scanning Electron microscopy

All lenticules were immediately cleaned with normal saline and immersed in 1.25% glutaraldehyde at 4 °C for 2 h. The specimens were treated with osmium acid before being dehydrated in a series of graded ethanol baths and then critical point dried. In each group, specimens from the odd-numbered rabbits were facing upward, and those from the even-numbered rabbits were facing downward. The samples were then mounted on scanning electron microscopy (SEM) stubs, sputter coated, and examined with via SEM (Hitachi S-SU8010).

### Surface quality analysis

Specimens were graded and scored by two observers (YZ and FZ) in a blinded fashion. The surface quality of the lenticules was assessed on the basis of the electron micrographs at 100× and 300× magnification. A scoring system that we have previously described [8] was used to evaluate surface characteristics of the lenticules. Four criteria were utilized to assess surface morphology: surface relief, regularity of the surface structure, the proportion of the surface that was irregular, and the position of the irregular area (Table 1). For surface relief, the entire lenticule was evaluated at 100× magnification. All other criteria were assessed at 300× magnification. A maximum of 16 points could be assigned to each lenticule.

**Table 1:** Criteria for evaluating surface characteristics

	Criterion and Magnification	Appearance	Scores
A	Surface relief ×100	Very smooth	4
		Smooth	3
		Rough	2
		Very rough	1
B	Regularity of surface structure ×300	Completely regular	4
		Almost Regular	3
		Partially regular	2
		Not regular	1
C	Portion of surface irregular ×300	< 10% of cut surface	4
		11–25% of cut surface	3
		26–50% of cut surface	2
		> 50% of cut surface	1
D	Position of the irregular area ×300	No irregularities	4
		Peripheral only	3
		Large region	2
		All over	1

### Statistical analysis

First, the normality of the paired difference was tested via the Kolmogorow-Smirnow test. Then, the paired t-test or the Wilcoxon matched-pairs signed-ranks test was used assess differences between groups. All analyses were performed in SPSS version 19.0 (SPSS Inc., Chicago, IL, USA), and *p* < 0.05 was considered statistically significant.

## Results

All surgical procedures were performed successfully and the lenticules were extracted smoothly. One myopia lenticule that was facing downward was handled failed in preparation for imaging, thus 15 lenticules were ultimately graded.

On the whole, 12 lenticules showed smooth surfaces at 100× magnification. At 300× magnification, regularly arranged tissue bridges were observed in more than 75% of the entire region—areas of disordered tissue bridges constituted less than 25% of the total area and were distributed peripherally. A total of 13 points was assigned to 11 specimens and 12 points was assigned to the other specimen.

Three lenticules exhibited with partial rough surfaces. In 1 hyperopia lenticule facing downward, less than 25% of the total area was irregular. In 1 hyperopia lenticule facing upward and 1 myopia lenticule facing downward, more than 25% of the total area was heterogeneous with irregularities distributed over a large region.

**Fig. 1:**
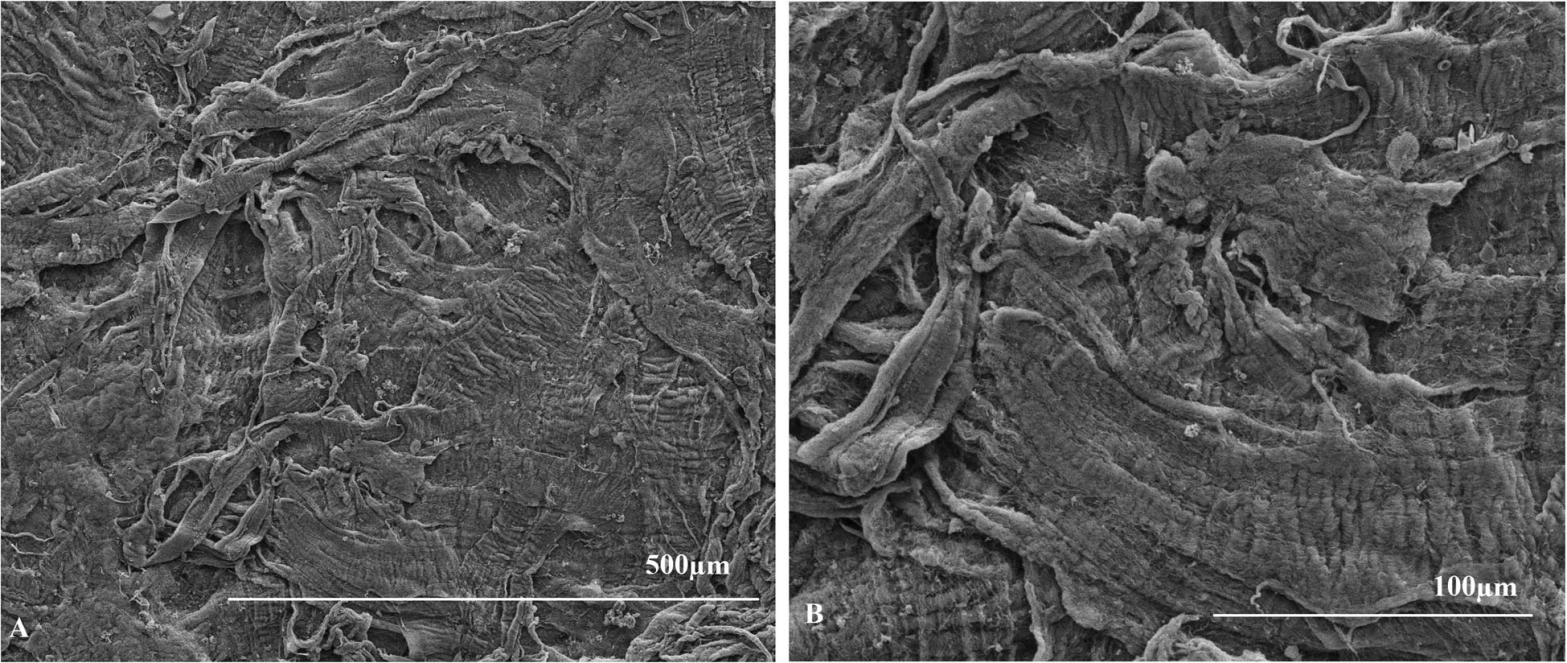
Images of anterior surface of hyperopia lenticule from the left eye of the rabbit numbered 3 at 100× magnification (**a**) and at 300 × magnification (**b**). With example of the score result of 8 points

**Fig. 2:**
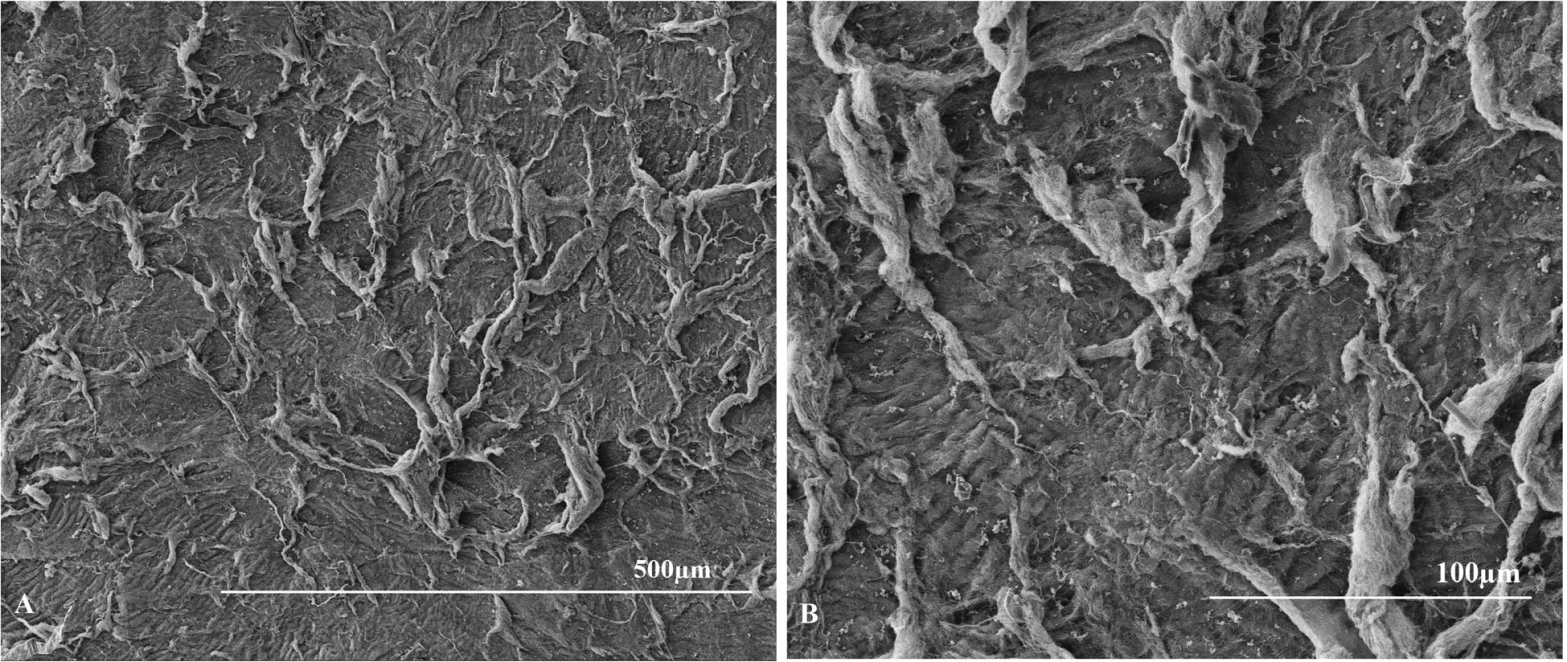
Images of anterior surface of hyperopia lenticule from the left eye of the rabbit numbered 7 at 100× magnification (**a**) and at 300 × magnification (**b**). With example of the score result of 13 points

**Fig. 3:**
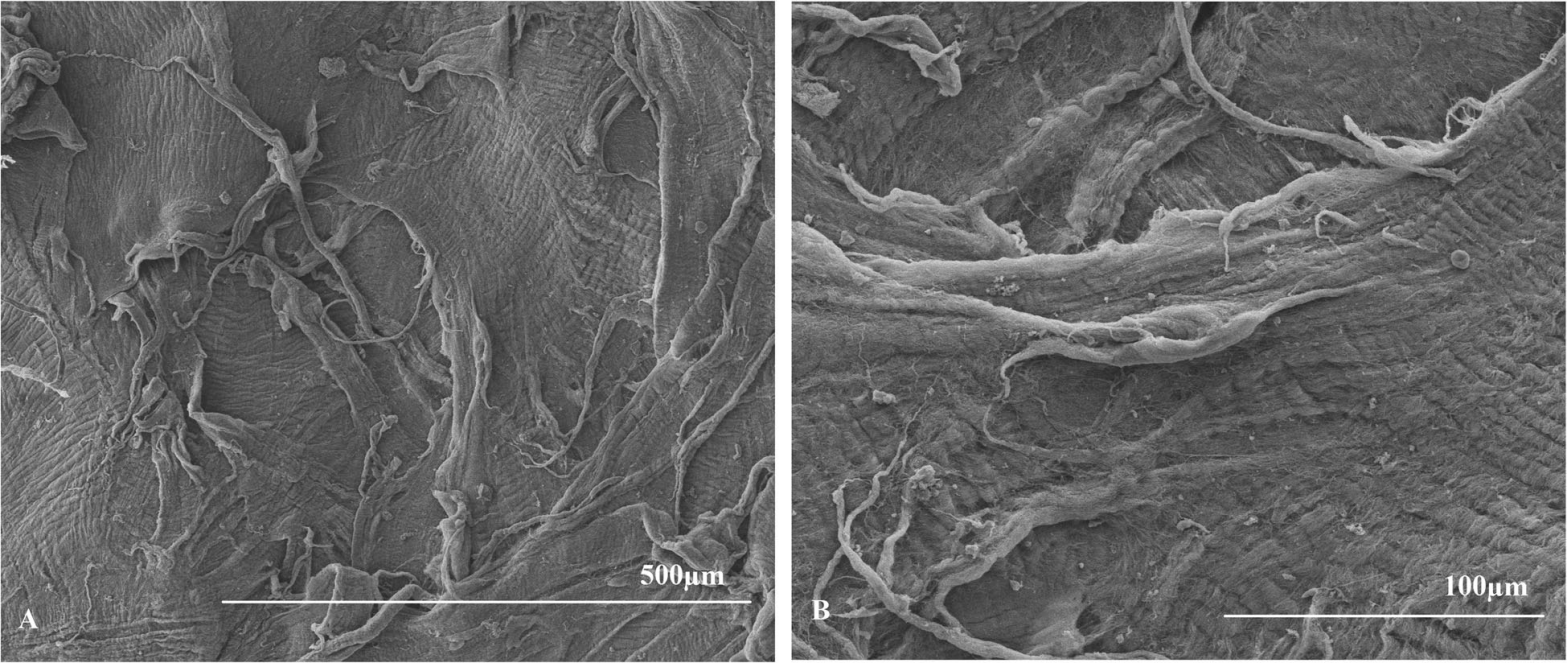
Images of posterior surface of hyperopia lenticule from the left eye of the rabbit numbered 2 at 100× magnification (**a**) and at 300 × magnification (**b**). With example of the score result of 10 points

**Fig. 4:**
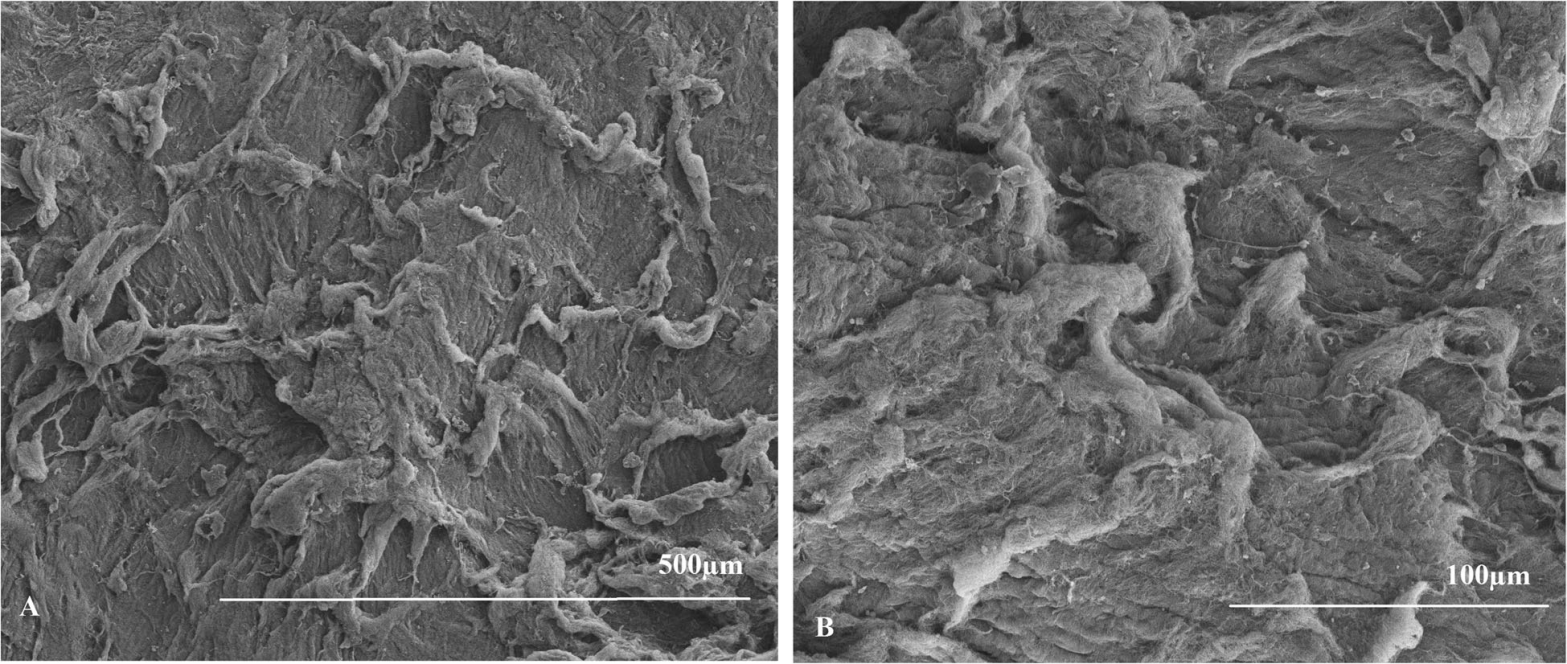
Images of posterior surface of myopia lenticule from the right eye of the rabbit numbered 2 at 100× magnification (**a**) and at 300 × magnification (**b**). With example of the score result of 8 points

The mean scores of anterior surfaces were 11.75 ± 2.50 for the hyperopia group and 12.75 ± 0.50 for the myopia group (Table 2). For the posterior surfaces, the average scores were 12.25 ± 1.50 for the hyperopia group and 11.33 ± 2.89 for the myopia group (Table 2). There were no significant differences between the lenticule surface scores of the two groups (*p* = 0.655 for the anterior surface and *p* = 0.317 for the posterior surface). Representative images of the lenticules are shown in (Figs. 1, 2, 3 and 4).

**Table 2:** Score results for lenticules in two groups

Surface	Group	Sample	Surface Relief	Regularity of Surface	Portion of Surface Irregular	Position of the Irregular	Score	Average	SD
Anterior	Hyperopia	1 L	3	3	4	3	13	11.75	2.50
		3 L	2	2	2	2	8		
		5 L	3	3	4	3	13		
		7 L	3	3	4	3	13		
	Myopia	1R	3	3	4	3	13	12.75	0.50
		3R	3	3	4	3	13		
		5R	3	3	3	3	12		
		7R	3	3	4	3	13		
Posterior	Hyperopia	2 L	2	2	3	3	10	12.25	1.50
		4 L	3	3	4	3	13		
		6 L	3	3	4	3	13		
		8 L	3	3	4	3	13		
	Myopia	2R	2	2	2	2	8	11.33	2.89
		6R	3	3	4	3	13		
		8R	3	3	4	3	13		

*SD* standard deviation

1 L means lenticule from the left eye of the rabbit numbered 1 and so on

## Discussion

Creating a lenticule with good surface quality is essential in SMILE, because it has been proved that lenticule quality is significantly correlated with the incidence of vision-threatening complications and refractive outcomes [9–12]. Therefore, it is important to guarantee lenticule surface quality in SMILE for hyperopia correction. In the current study, we evaluated lenticule surface characteristics after SMILE for hyperopia correction in rabbits for the first time [13].

Tissue bridges were distributed evenly in most areas of the lenticules. It has been reported that tissue bridges were the main entity for surface irregularities on the corneal lenticule surface [14]. It was described as residual fibers between the interfaces after completion of laser cuts. Laser cutting, as well as surgical manipulation, could affect the arrangement of tissue bridges. In the current study, although the laser settings were exactly the same as those used in the previous study evaluating human lenticules, tissue bridges in rabbit lenticules were longer and looser compared to those of human lenticules [8]. There are two potential reasons for this. One is that the structure of corneal collagen fibers in rabbits may differ from that of humans. Another possibility is that the rabbits had bad coordination during the operation and required more surgical dissection, which may have affect the surface quality and cause the roughness.

The lenticules exhibited comparable surface characteristics in both of the groups in the study, and the central and periphery regions of the posterior surfaces of the hyperopia lenticules exhibited similar arranged tissue bridges. However, rough lenticules occurred in twice as many lenticules in the hyperopia group compared to the myopia group, and the results implied that the hyperopia lenticule may be more likely to presented with roughness. With regard to the treatment of hyperopia, it has been reported that lenticule shape was thicker in the periphery and thinner in the center [15, 16]. This may increase the difficulty of surgical manipulation and result in surface roughness. To reduce operative difficulty, the minimal central thickness of hyperopic lenticule was limited to 25 μm in the current study. Also, the surgeon had extensive experience in the performance of SMILE and performed the procedure gently, and tried his utmost not to induce surface roughness artificially. Notably however, among the 3 cases that exhibited partially rough surfaces, in 2 cases it was related to the posterior surface and in only 1 case it was related to the anterior surface The results suggested that dissection between the lenticule-stromal bed interface could be more difficult than dissection at the cap-lenticule interface: the anterior surface can be dissected under the cap smoothly, but for the posterior surface, dissection was done more slowly and involved repetition action because the lenticule was thin and unfixed. This emphasizes the importance of good cooperation and careful surgical manipulation during the procedure, for both hyperopia and myopia correction [17].

Visible cavitation holes were absent in all lenticules except 1 in the myopia group that exhibited a few cavitation holes on the anterior surface. Cavitation holes are gas bubbles formed during the vaporization of the corneal tissue, and they affect scanning quality [14]. The scanning quality of the both surfaces of the lenticules was comparable in the two groups. The working principle of the femtosecond laser may explain this finding. The femtosecond laser is a near-infrared laser that produces ultra-short pulses of light [18]. In the non-thermal ablation process that is achieved via corneal photodisruption, a plasma state develops with optical breakdown, and some cavitation gas bubbles are formed. A series of bubbles is created resulting in separating of the corneal tissue at a precise depth [19]. The system used in the procedure could accomplish homogeneous cut in most cases. However, in some unexpected cases, for example, corneal edema or eye rotation during the laser scanning, may affect the homogeneity of laser cutting and result in cavitation holes.

Previous studies had investigated the corneal surface characteristics in myopia correction during SMILE, and reported that pulse energy and laser frequency are two foremost parameters influencing scanning quality [20]. Heichel [14] firstly reported scoring of lenticule surface quality in porcine corneas using the original VisuMax femtosecond laser system with a repetition rate of 200 kHz and a pulse energy of 185 nJ. Although lenticules of predictable surface quality were created in the procedure, the results suggested that laser settings should be improved. Kunert [10] evaluated lenticule surface characteristics with a fixed repetition rate of 200 kHz and different energy levels (150, 180, and 195 nJ). The highest surface regularity score was achieved using the lowest pulse energy, presenting that lower pulse energy facilitates a smoother cut surface. Furthermore, smoother lenticule surfaces were reported observed using a higher frequency laser [8, 21].

Based on the aforementioned investigations, a new generation VisuMax laser with settings of a 500 kHz repetition rate and a pulse energy of 130 nJ was investigated. Researchers studied the scan quality of corneal lenticules in the context of myopia treatment using the new laser system, and found that both sides of the lenticules exhibited smooth surfaces [21]. Therefore, in the current study, the exact same laser settings were used to perform hyperopia correction in SMILE, and enabled homogeneous cutting.

The current study had some limitations, the foremost being the small number of specimens involved. Also, while precautions were taken during preparation for imaging, scratches and grooves may have been generated. Lastly, the refractive outcomes, were not evaluated in the current study, need further investigations.

## Conclusions

In conclusion, scan quality of lenticules after SMILE for hyperopia correction is comparable to that of myopia lenticules. The shape of hyperopic lenticule may increase the difficulty of surgical manipulation and result in surface roughness.

## Data Availability

All data used and analyzed in this study are available upon request from the first author; Yu Zhao.

## Abbreviations

SMILE: Small incision lenticule extraction
LASEK: Laser epithelial keratomileusis
LASIK: Laser in situ keratomileusis
SEM: Scanning electron microscopy
